# Two Novel 1,4‐Naphthoquinone Derivatives as Potent Agents Against Multidrug‐Resistant *Staphylococcus aureus* and Pathogenic Gram‐Positive Bacteria

**DOI:** 10.1002/ddr.70353

**Published:** 2026-07-20

**Authors:** HyunChan Song, Haena Lee, Ji‐Hyun Yoon, Hyunkyung Choi, Hakwon Kim, Ki‐Young Kim

**Affiliations:** ^1^ Graduate School of Biotechnology Kyung Hee University Yongin Republic of Korea; ^2^ Department of Applied Chemistry, Global Center for Pharmaceutical Ingredient Materials Kyung Hee University Yongin Republic of Korea

**Keywords:** antimicrobial resistance, naphthoquinone, topical agents

## Abstract

Drug‐resistant bacteria such as Methicillin‐resistant *Staphylococcus aureus* (MRSA) and Quinolone‐resistant *S. aureus* (QRSA), are a growing problem, creating a need for the development of novel antimicrobial agents. In this study, we designed and synthesized two novel 1,4‐naphthoquinone derivatives, **LHN‐1034** and **LHN‐1035,** and evaluated their antibacterial efficacy. Both compounds exhibited great antimicrobial activity against a group of Gram‐positive bacteria, including MRSA and QRSA, with Minimum Inhibitory Concentrations (MICs) ranging from 50 to 100 µM. Mechanism studies revealed that the antibacterial effect is oxygen‐dependent. The compounds act as redox‐cycling agents that induce intracellular reactive oxygen species (ROS), which disrupts cell membrane integrity and leads to cell lysis. In silico assessments predict low intestinal absorption and skin permeability, suggesting these compounds are suited for topical application. In conclusion, **LHN‐1034** and **LHN‐1035** are candidates for developing topical agents against resistant Gram‐positive infections.

## 1 Introduction

The rise of antimicrobial resistance (AMR) is a serious global health problem, leading to significant sickness and mortality (Murray et al. 2022). Within this broader challenge, the emergence and spread of multidrug‐resistant (MDR) bacterial strains is of particular concern (Alara and Alara 2024). These strains have innate or acquired resistance to multiple classes of antibiotics, severely limiting therapeutic options and complicating patient management (Catalano et al. 2022). Important among these are Methicillin‐resistant *Staphylococcus aureus* (MRSA) and Quinolone‐resistant *S. aureus* (QRSA) (Stefani et al. 2012; Tanaka et al. 2000). MRSA, a well‐known pathogen, is resistant not only to methicillin but often to other beta‐lactam antibiotics, making common staphylococcal infections difficult to treat (Dilawer Issa and Dlshad Muhsin 2024). While MRSA has been extensively studied, the increasing emergence of QRSA, which exhibits resistance to quinolone, further limits the effective treatment options for *S. aureus* (Hooper 2003). The emergence of *S. aureus* strains resistant to both methicillin and quinolones presents an even greater clinical challenge, requiring a deeper understanding of their resistance mechanisms and effective control strategies (Piewngam and Otto 2024).

The impact of uncontrolled multidrug‐resistant (MDR) bacteria extends far beyond the treatment outcomes of individual patients, posing a serious threat to the foundations of modern medicine (Laxminarayan et al. 2013). If these problems are left unaddressed, they could lead to a post‐antibiotic era where every day medical procedures, like surgeries, organ transplants, and cancer treatments, become extremely risky due to untreatable infections (Teillant et al. 2015; World Health Organization 2014). This would not only cause a significant increase in morbidity and mortality worldwide, potentially causing millions of deaths each year, but also place a tremendous burden on healthcare systems through longer hospital stays, increased demand for intensive care, and massive increases in treatment costs (Murray et al. 2022; World Health Organization 2014). In 2019, antimicrobial resistance (AMR) was associated with an estimated 4.95 million deaths globally, with *S. aureus* being one of the leading pathogens responsible for these fatalities (Murray et al. 2022). The economic burden is equally staggering, leading to 100 trillion dollars in increased healthcare costs until 2025, due to prolonged hospitalizations and the requirement for intensive care (Pulingam et al. 2022). Current treatment options for MRSA infections primarily rely on last‐resort antibiotics such as glycopeptides and lipopeptides (Hines et al. 2020). In antimicrobial susceptibility studies, ciprofloxacin, a broad‐spectrum fluoroquinolone, is frequently used as a standard drug to evaluate the relative efficacy and resistance profiles of novel DNA gyrase‐targeting agents (Sharma et al. 2010). Furthermore, the spread of MDR strains can seriously affect farming and food production, as antibiotics are also used in animals, threatening global food security and economic stability (Tang et al. 2017).

Therefore, these challenges emphasize the urgent need for novel antimicrobial candidates with distinct mechanisms of action (Breijyeh and Karaman 2023). In this context, quinone derivatives, which are known to exert anticancer, antibacterial, and antimalarial activities through the generation of reactive oxygen species (ROS), have emerged as valuable scaffolds for drug development (Dahlem Junior et al. 2022).

Quinones are a class of organic compounds derived from aromatic rings, in which two carbon atoms are oxidized to form carbonyl groups, typically arranged within a conjugated system (Wendlandt and Stahl 2015). They are well known for their redox activity, serving as electron carriers in biological processes such as cellular respiration and photosynthesis (Wendlandt and Stahl 2015). Their capacity to generate reactive oxygen species (ROS) underlies their diverse biological activities, including antibacterial, anticancer, and antimalarial effects (Zhang et al. 2021). These properties make quinones valuable scaffolds in drug development and medicinal chemistry (Zhang et al. 2021). Among the diverse quinone family, 1,4‐naphthoquinone possesses ketone groups at the 1,4‐positions that facilitate electron transfer and stabilize radical intermediates. This compound exhibits unique antimicrobial activity by serving as an efficient electron acceptor and strongly inducing ROS generation through effective intracellular redox cycling (Navarro‐Tovar et al. 2023). Furthermore, the ease of introducing substituents at multiple positions enables extensive structural diversification. In particular, halogenation of the 1,4‐naphthoquinone core has been reported to significantly enhance antimicrobial properties (Navarro‐Tovar et al. 2023).

**FIGURE 1:**
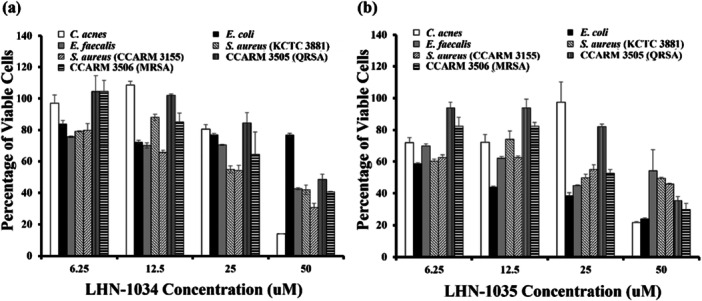
Percentage of viable cells after chemical treatment. (a) The activity of mitochondrial dehydrogenases in viable cells compared to the control after treating **LHN‐1034**. (b) The activity of mitochondrial dehydrogenases in viable cells compared to the control after treating **LHN‐1035**.

YM155 (sepantronium bromide; Supporting Information S1: Figure 1), a naphthoquinone–imidazolium fused compound originally developed as an anticancer candidate, was reported to suppress survivin and exhibit anticancer activity (Nakahara et al. 2007). In addition, YM155 was found to display antibacterial activity against methicillin‐resistant *Staphylococcus aureus* (MRSA), which prompted further efforts to generate antibacterial derivatives through structural modification (Jang et al. 2019). In a previous study, various derivatives were synthesized by introducing substituents at the N3 position of YM155, among which compound C5 (Supporting Information S1: Figure 1b), bearing a hexyl substituent at the N3 position, exhibited enhanced antibacterial activity against MRSA compared with YM155 (Jang et al. 2019). However, C5 showed limited activity against Gram‐negative bacteria because of the outer membrane barrier, and its antibacterial activity was associated with oxygen‐dependent ROS generation (Jang et al. 2019).

These literature findings provided a structural rationale for further optimization and mechanistic investigation of the naphthoquinone scaffold. Accordingly, in the present study, we designed and synthesized novel naphthoquinone derivatives bearing a nitro group at the 5‐ or 8‐position and a primary amine at the 2‐ or 3‐position to overcome these limitations. We aimed to evaluate the antimicrobial efficacy of these two novel compounds, LHN‐1034 and LHN‐1035, against a diverse panel of microorganisms, including several drug‐resistant bacteria such as MRSA and QRSA. Particular attention was given to their effects against *S. aureus*, *Cutibacterium acnes*, and *Enterococcus faecalis*, all of which are clinically important human pathogens.

## 2 Materials and Methods

### 2.1 Chemical

All glassware was thoroughly dried in a convection oven. Reactions were monitored using thin‐layer chromatography (TLC) with commercial silica gel 60 F254 (Merck KGaA, Germany), and spots were visualized under UV light at 254 or 365 nm. Products were purified by flash column chromatography using 230‐400 mesh ASTM silica gel (Merck KGaA, Germany) or by recrystallization with combinations of various solvents (Still et al. 1978). Extra pure‐grade solvents for column chromatography were purchased from Samchun Chemicals (Korea) and Duksan Chemicals (Korea). ^1^H and ^13^C NMR spectra were recorded on a JEOL JNM‐ECZ400S (at 400 MHz for ^1^H NMR and 100 MHz for ^13^C NMR). In the ^1^H NMR spectra chemical shifts are reported in parts per million (ppm) relative to tetramethylsilane (TMS), and coupling constants are given in Hertz (Hz). Splitting patterns are indicated as follows: s, singlet; d, doublet; dd, doublet of doublet; t, triplet; q, quartet; and m, multiplet. ^13^C NMR spectra are reported in ppm, referenced to CDCl_3_ and DMSO‐*d*_*6*_. Melting points (m.p.) were determined on a Barnstead Electrothermal 9100 instrument and are uncorrected. All chemical reagents were acquired (Acros Organics, USA; Sigma‐Aldrich, USA; and TCI, Japan) and were used as received.

#### 2.1.1 2,3‐Dibromonaphthalene‐1,4‐dione (**1**)

A solution of 1,4‐naphthoquinone (2.00 g, 12.7 mmol) in glacial acetic acid (20.0 mL) was treated dropwise with Br_2_ (1.43 mL, 27.8 mmol). The mixture was stirred at room temperature for 1 h, then heated under reflux for 19 h. Upon completion, the reaction mixture was poured into ice–water (250 mL), producing a precipitate, which was collected by filtration, washed with water, and dried under vacuum. The crude product was purified by recrystallization from EtOH (60.0 mL). The resulting solid was collected by filtration, washed with EtOH, and air‐dried to afford the desired yellow solid (3.70 g, 91.3%).^1^H NMR (CDCl_3_, 400 MHz): 8.20 (*dd*, *J* = 3.4, 5.8 Hz, 2H), 7.79 (*dd*, *J* = 3.4, 5.8 Hz, 2H); ^13^C NMR (CDCl_3_, 100 MHz): 175.84, 142.58, 134.53, 130.79, 128.23.

#### 2.1.2 2,3‐Dibromo‐5‐nitronaphthalene‐1,4‐dione (**2**)

A solution of 2,3‐dibromonaphthalene‐1,4‐dione (0.500 g, 1.58 mmol) in sulfuric acid (10.0 mL) was treated at 0°C with a solution of sodium nitrate (0.874 g, 10.3 mmol) in sulfuric acid (3.00 mL), added in two portions. The ice bath was then removed, and the mixture was allowed to warm to room temperature over 30 min, followed by stirring for an additional 30 min. The mixture was subsequently heated to 40°C and stirred for 3 h. Upon completion, the reaction mixture was cooled to room temperature, poured into ice–water (100 mL), and extracted with CH_2_Cl_2_. The organic layer was separated, washed with brine, and dried over Na_2_SO_4_. After filtration, the solvent was removed under reduced pressure, and the residue was purified by silica gel flash column chromatography (ethyl acetate/hexane = 1:4–1:2) to afford the desired yellow solid (0.340 g, 60.4%).^1^H NMR (CDCl_3_, 400 MHz): 8.40 (*dd*, *J* = 1.2, 8.0 Hz, 1H), 7.94 (*t*, *J* = 7.8 Hz, 1H), 7.78 (*dd*, *J* = 1.2, 8.0 Hz, 1H); ^13^C NMR (CDCl_3_, 100 MHz): 174.09, 172.52, 149.20, 142.56, 142.03, 135.32, 131.60, 130.43, 128.13, 121.90.

#### 2.1.3 Synthesis of N‐Boc‐naphthoquinone Derivatives (**3a, 3b**)

A solution of 2,3‐dibromo‐5‐nitronaphthalene‐1,4‐dione (0.233 g, 0.645 mmol) in EtOH (6.40 mL) was treated with *N*‐BOC‐1,6‐diaminohexane (0.290 mL, 1.29 mmol) at room temperature. The mixture was stirred for 3 h. Upon completion, the reaction mixture was diluted with CH_2_Cl_2_, washed with water and brine, and the organic layer was separated and dried over Na_2_SO_4_. After filtration, the solvent was removed under reduced pressure, and the residue was purified by silica gel flash column chromatography (ethyl acetate/hexane = 3:7) to afford the desired products, **3a** and **3b**.

##### 2.1.3.1 Tert‐butyl 6‐(3‐bromo‐8‐nitro‐1,4‐dioxo‐1,4‐dihydronaphthalen‐2‐ylamino)hexylcarbamate (**3a**)

Red solid (0.156 g, 48.7%); ^1^H NMR (DMSO‐*d*_*6*_, 400 MHz): 8.18 (*d*, *J* = 7.6 Hz, 1H), 8.04 (*dd*, *J* = 0.8, 8.0 Hz, 1H), 7.97 (*t*, *J* = 8.0 Hz, 1H), 7.64 (*bs*, 1H), 6.73 (*t*, *J* = 5.2 Hz, 1H), 3.64 (*s*, 2H), 2.88 (*q*, *J* = 6.4 Hz, 2H), 1.57 (*t*, *J* = 6.8 Hz, 2H), 1.35 (*s*, 11H), 1.26‐1.25 (*m*, 4H); ^13^C NMR (DMSO‐*d*_*6*_, 100 MHz): 177.42, 173.38, 155.58, 147.80, 135.75, 132.81, 128.58, 126.38, 77.29, 44.14, 30.61, 29.39, 28.26, 25.93, 25.75.

##### 2.1.3.2 Tert‐butyl 6‐(3‐bromo‐5‐nitro‐1,4‐dioxo‐1,4‐dihydronaphthalen‐2‐ylamino)hexylcarbamate (**3b**)

Red oil (0.155 g, 48.3%); ^1^H NMR (DMSO‐*d*_*6*_, 400 MHz): 8.15 (*dd*, *J* = 1.2, 7.6 Hz, 1H), 8.02 (*dd*, *J* = 1.2, 8.0 Hz, 1H), 7.88 (*t*, *J* = 7.8 Hz, 1H), 6.73 (*t*, *J* = 5.4 Hz, 1H), 3.71 (*q*, *J* = 7.1 Hz, 2H), 2.88 (*q*, *J* = 6.5 Hz, 2H), 1.60 (*t*, *J* = 7.0 Hz, 2H), 1.35 (*s*, 11H), 1.27‐1.25 (*m*, 4H); ^13^C NMR (DMSO‐*d*_*6*_, 100 MHz):178.09, 171.78, 155.57, 147.81, 133.64, 131.43, 128.85, 127.95, 122.27, 77.28, 44.28, 30.50, 29.41, 28.25, 25.93, 25.76.

#### 2.1.4 Synthesis of HCl‐Salt of Naphthoquinone Derivatives (**LHN‐1034**, **LHN‐1035; 4a, 4b**)

A solution of the *N*‐Boc‐naphthoquinone derivative (1 equiv, **3a** or **3b**) in EtOAc (0.1 M) was treated with HCl (1 M in ethyl acetate, 10 equiv) at room temperature. The mixture was stirred for 12 h, after which the solvent was removed under reduced pressure. The crude product was purified by recrystallization from ethyl acetate. The resulting solid was collected by filtration, washed with ethyl acetate, and air‐dried to afford the desired product.

##### 2.1.4.1 6‐(3‐Bromo‐8‐nitro‐1,4‐dioxo‐1,4‐dihydronaphthalen‐2‐ylamino)hexan‐1‐aminium chloride (**LHN‐1034; 4a**)

The compound was prepared from **3a** as a red solid (0.178 g, 92.5%); m.p. 237.6 ‐ 238.1°C; ^1^H NMR (DMSO‐*d*_*6*_, 400 MHz): 8.18 (*dd*, *J* = 1.6, 7.6 Hz, 1H), 8.04‐7.97 (*m*, 2H), 7.86 (*bs*, 3H), 7.73 (*t*, *J* = 6.0 Hz, 1H), 3.65 (*q*, *J* = 6.9 Hz, 2H), 2.74 (*t*, *J* = 7.2 Hz, 2H), 1.62‐1.50 (*m*, 4H), 1.31‐1.29 (*m*, 4H); ^13^C NMR (DMSO‐*d*_*6*_, 100 MHz): 177.63, 147.75, 135.95, 133.07, 128.25, 126.34, 43.86, 38.65, 30.63, 26.89, 25.60, 25.53; HRMS (FAB) m/z Calcd. for C_16_H_19_BrClN_3_O_4_ [M‐Cl]^+^ 396.0553, found 396.0551.

##### 2.1.4.2 6‐(3‐Bromo‐5‐nitro‐1,4‐dioxo‐1,4‐dihydronaphthalen‐2‐ylamino)hexan‐1‐aminium chloride (**LHN‐1035; 4b**)

The compound was prepared from **3b** as a red solid (0.128 g, 73.0%); m.p. 183.4 ‐ 184.6°C; ^1^H NMR (DMSO‐*d*_*6*_, 400 MHz): 8.15 (*dd*, *J* = 1.0, 7.8 Hz, 1H), 8.04 (*dd*, *J* = 1.2, 8.0 Hz, 1H), 7.91‐7.86 (*m*, 5H), 3.70 (*q*, *J* = 7.2 Hz, 2H), 2.74 (*s*, 2H), 1.62‐1.52 (*m*, 4H), 1.31 (*t*, *J* = 3.4 Hz, 4H); ^13^C NMR (DMSO‐*d*_*6*_, 100 MHz): 179.02, 148.25, 134.32, 131.98, 129.31, 128.88, 128.60, 123.11, 44.42, 39.19, 31.13, 27.43, 26.15, 26.07; HRMS (FAB) m/z Calcd. for C_16_H_19_BrClN_3_O_4_ [M‐Cl]^+^ 396.0553, found 396.0558.

### 2.2 Strain Preparation

All bacterial strains used in this study are listed in Table 1. *Salmonella typhimurium*, *Acinetobacter baumannii*, *Escherichia coli*, *E. faecalis*, and *S. aureus* [KCTC 3881, CCARM 3155, CCARM 3505 (QRSA), and CCARM 3506 (MRSA)] were routinely cultured in Tryptic Soy Broth (TSB) or on Tryptic Soy Agar (TSA) at 37°C under aerobic conditions (Fisher and Phillips 2009; Koutsoumanis et al. 2004; Peleg et al. 2008; Sezonov et al. 2007; Valero et al. 2009). *Gardnerella vaginalis* [KCTC 5096] was grown in mBHI broth at 37°C under anaerobic condition (Catlin 1992). *C. acnes* [CCARM 0081] was grown in Reinforced Clostridial Medium (RCM) at 37°C under anaerobic conditions (Achermann et al. 2014).

**TABLE 1:** Bacterial strains and culture conditions used in this study.

Organism	Strain designation	Growth medium	Incubation temperature	Atmosphere
*Salmonella Typhimurium*	CCARM 0240	TS	37°C	Aerobic
*Acinetobacter baumannii*	KACC 12454	TS	37°C	Aerobic
*Gardnerella vaginalis*	KCTC 5096	mBHI	37°C	Anaerobic
*Cutibacterium acnes*	CCARM 0081	RCM	37°C	Anaerobic
*Escherichia coli*	CCARM 0237	TS	37°C	Aerobic
*Enterococcus faecalis*	CCARM 5511	TS	37°C	Aerobic
*Staphylococcus aureus*	KCTC 3881	TS	37°C	Aerobic
*Staphylococcus aureus*	CCARM 3155	TS	37°C	Aerobic
*Staphylococcus aureus*	CCARM 3505 (QRSA)	TS	37°C	Aerobic
*Staphylococcus aureus*	CCARM 3506 (MRSA)	TS	37°C	Aerobic

All bacterial cultures were prepared from frozen 20% glycerol stocks stored at ‐80°C. For experimental procedures, fresh overnight cultures were prepared by inoculating a single colony into the respective liquid medium and incubating under the specified conditions.

### 2.3 Antimicrobial Susceptibility Testing

The Minimum Inhibitory Concentration (MIC) of compounds was determined for all bacterial strains listed in Table 1 using a twofold micro‐broth dilution method in accordance with the guidelines established by the Clinical and Laboratory Standards Institute (CLSI) document M100 (Clinical and Laboratory Standards Institute 2024; Nguyen and Kim 2020; Song et al. 2022).

Briefly, fresh overnight cultures of each bacterial strain were adjusted to a turbidity equivalent to a 0.5 McFarland standard, which corresponds to approximately 1 − 2 × 10^8^ colony‐forming units (CFU)/mL. This inoculum was then diluted to achieve a final concentration of 5 × 10^5^ CFU/mL in each well of a sterile 96‐well microtiter plate. Each well received a 100 µL volume of the adjusted bacterial suspension. Test compounds were serially diluted twofold across the wells, ranging from an initial concentration of 100 µM down to 0.156 µM, with 100 µL added to each well. Growth control wells containing only bacteria, and sterility control wells containing only medium, were included in each plate.

Plates were incubated under appropriate growth conditions (Table 1) for 48 h. After incubation, the MIC was visually determined as the lowest concentration of the compound that completely inhibited visible growth. All experiments were performed in triplicate on separate occasions.

### 2.4 Bacteria Viability Assay

The viability of the bacterial strains upon treatment with test compounds was quantified using the EZ‐CYTOX Cell Viability Assay Kit (DoGenBio Co. Ltd., Seoul, South Korea) (Ishiyama et al. 1993). This colorimetric assay measures the activity of mitochondrial dehydrogenases in viable cells, which convert the tetrazolium salt WST‐8 into a water‐soluble formazan dye, correlating directly with cell viability. Cultured bacterial strains were adjusted to a 5 × 10^5^ CFU/mL. 100 µL of the adjusted cell suspension were dispensed into each well of a sterile 96‐well microtiter plate. Test compounds were then added to the wells at various concentrations in a twofold serial dilution from a maximum concentration of 100 µM. Control wells included untreated cells and medium‐only wells.

After incubation for 48 h under the specific conditions detailed for each strain in Table 1, 10 µL of the EZ‐CYTOX reagent was added to each well. The plates were then incubated for an additional 1–4 h at the respective incubation temperature. The absorbance was then measured at 450 nm using a microplate spectrophotometer (Epoch, BioTek Instruments Inc., Winooski, VT, USA). The absorbance values from the blank control wells were subtracted from all experimental wells. Cell viability was calculated as a percentage relative to the untreated control cells. All experiments were performed in triplicate on separate occasions.

### 2.5 Thiobarbituric Acid‐Reactive Substances (TBARS) Assay

The thiobarbituric acid‐reactive substances (TBARS) assay was conducted to measure malondialdehyde (MDA) with a slight change from previous study (Buege and Aust 1978). Bacterial cells were treated with **LHN‐1034** and **LHN‐1035** at the concentration of 2 µM and 20 µM. Cells were incubated for 2 h at 37°C. Cells were then centrifuged at 12,000 rpm for 10 min, and cell pellets were resuspended with lysis buffer (pH8.0, 10 mM Tris–HCl, 1 mM EDTA, 100 mM NaCl, 2% Tri‐ton X‐100, and 1% SDS). Cells were then sonicated on ice using an ultrasonic sonicator (Sonics & materials Inc, USA) for total sonication of 10 min at amplitude of 60%, interval of 10 s of sonication and 10 s of cooling followed by centrifugation. The supernatant was mixed with thiobarbituric acid in 5% trichloroacetic acid. The mixture was heated at 95°C for 30 min. After heating all samples kept on ice, before measuring absorbance at 532 nm. All experiments were performed in triplicate on separate occasions.

### 2.6 Spotting Analysis

To compare the growth differences under aerobic and anaerobic conditions on agar plate, spotting analysis was done (Miles et al. 1938). *S. aureus* (KCTC 3881) was cultured for overnight in TSB before the assay. The medium was replaced to fresh medium and incubated for an additional 2 h. Cells were then diluted to an absorbance of 0.05 at 600 nm. Cell suspension was dispensed into each well of a 96‐well microplate. After diluting each compound to an indicated concentration, cells and compounds were applied to TSA plates and incubated under aerobic or anaerobic conditions. All plates were imaged after 48 h of incubation. All experiments were performed in triplicate on separate occasions.

### 2.7 Cell Lysis Assay

To assess dose‐dependent cell lysis, bacterial cells were harvested from overnight cultures and washed twice with sterile phosphate‐buffered saline (PBS, pH 7.4) to remove residual medium components and stop further growth (Chen and Cooper 2002). Cells were then resuspended in PBS to an optical density at 600 nm of 0.5.

Cell suspensions were then treated with indicated concentrations of the test compound, ranging from 100 to 6.25 µM. After a 1 h incubation at 37°C, samples were centrifuged to pellet intact cells. The supernatant containing released cellular components was then collected. For reference, samples containing of each concentration of compound dissolved in PBS without cells were prepared and incubated simultaneously. This was done to ensure that the compound itself did not exhibit any absorbance at the wavelengths used for measurement, thereby to prevent false positives.

The amount of cell lysate released due to membrane damage was quantified by measuring the optical density (OD) of the supernatant at 260 nm using a microplate spectrophotometer (Epoch, BioTek Instruments Inc., Winooski, VT, USA). This wavelength represents leakage (260 nm). The OD values were corrected by subtracting the corresponding OD values obtained from the negative control wells for each compound concentration. All experiments were performed in triplicate on separate occasions.

### 2.8 DNA Gyrase Assay

The DNA Gyrase inhibitory activity of **LHN‐1034** and **LHN‐1035** was measured using a DNA gyrase assay kit (KOMA Biotech, Seoul, Korea) according to the instruction of manufacturer (Barrett et al. 1993).

DNA gyrase from *E. coli* was treated with **LHN‐1034** and **LHN‐1035** at 10 µM. Control groups included a positive control containing only the enzyme, a negative control without the enzyme, and a reference inhibitor group treated with ciprofloxacin. The mixtures were incubated at 37°C for 30 min before the addition of stop solution to terminate the assay. The reaction product was electrophoresed on a 1% agarose gel, and the bands were confirmed using EtBr staining. Linear kinetoplast DNA (kDNA) and decatenated kDNA were used as markers for analysis of the experimental results. Each band was quantified using ImageJ (NIH, Maryland, USA). All experiments were performed in triplicate on separate occasions.

### 2.9 Molecular Docking and Scoring Protocol

Molecular docking analyses were conducted to evaluate the binding characteristics of the synthesized naphthoquinone derivatives (**LHN‐1034** and **LHN‐1035**) within the DNA gyrase A (GyrA) A‐site. The GyrA–DNA–ligand ternary complex from *Mycobacterium tuberculosis* (PDB ID: 5BTC) was employed as the docking model, as it contains the conserved A‐site metal–DNA chelation pocket that serves as the canonical binding region for fluoroquinolone antibiotics (Spencer and Panda 2023). Because this pocket is highly conserved across bacterial species, including *S. aureus* and *E. coli*, 5BTC was considered an appropriate representative structure for comparative GyrA binding analysis.

The receptor structure was prepared by retaining only the essential components required for metal‐mediated binding, including the central Mg^2+^ ion, while removing non‐coordinating water molecules and the counter ion (Cl^−^). The binding site was defined as a sphere with an 11 Å radius centered on the co‐crystallized fluoroquinolone at the GyrA A‐site. **LHN‐1034** and **LHN‐1035** were constructed in three dimensions, protonated to their predominant ionic states at physiological pH 7.4, and energy‐minimized prior to docking (Petukh et al. 2013).

Docking simulations were performed using the LibDock algorithm implemented in BIOVIA Discovery Studio (Dassault Systèmes, San Diego, CA, USA) (Rao et al. 2007). For each ligand, up to 100 candidate binding poses were generated and initially ranked according to the LibDock score. To enhance the reliability of pose evaluation, additional scoring functions (LigScore1, LigScore2, PLP1, PLP2, Jain, PMF, and PMF04) were applied (Krovat and Langer 2004). Because these scoring functions operate on different numerical scales, all scores were normalized to Z‐scores, and a Combined Z‐score was calculated by summing the standardized values. This consensus scoring strategy reduces algorithm‐specific bias and facilitates the identification of the most credible binding conformations. For each ligand, the pose with the highest Combined Z‐score was selected as the representative docking conformation for subsequent interaction analysis and figure generation.

### 2.10 In Silico Toxicity Prediction

Putative toxicity‐related properties of **LHN‐1034** and **LHN‐1035** were evaluated using machine‐learning–based in silico prediction tools, including pkCSM and ProTox‐II (Pires et al. 2015; Banerjee et al. 2018). These platforms estimate organ toxicity, cardiotoxicity, and mutagenicity parameters based on chemical structure information. All predictions were performed using default parameters, and the results were interpreted as qualitative indicators for preliminary risk assessment rather than definitive safety evaluations.

### 2.11 Statistics

All experiments were performed at least three times, and data are presented as the mean ± S.D. Statistical analysis was performed using Microsoft Excel 2021 (Microsoft, Washington, USA) and Statistical Package for the Social Sciences (SPSS) 28.0 (IBM Corp, USA). Data were analyzed using Student's *t*‐test for comparisons between two groups or one‐way analysis of variance (ANOVA) followed by Dunnett's post hoc test for multiple comparisons against the control group. All graphs were analyzed through Microsoft Excel 2021.

## 3 Results

### 3.1 Chemical Synthesis

**Scheme 1:**
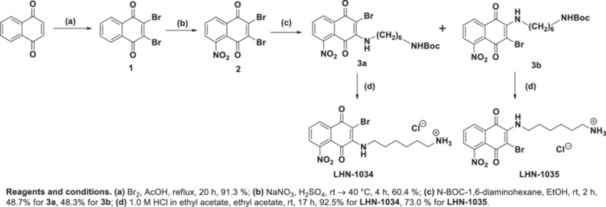
Synthesis of 5‐or 8‐nitro and 3‐bromo‐2‐aminonaphthoquinone derivatives (**LHN‐1034**, **LHN‐1035**).

The synthesis of the target derivatives was initiated from 1,4‐naphthoquinone (Scheme 1). Dibromo intermediate **1** was prepared through bromination with Br_2_ to facilitate amine substitution. Subsequent nitration at the C‐5 position using NaNO_3_ and concentrated H_2_SO_4_ yielded intermediate **2** and its regioisomer, 2,3‐dibromo‐6‐nitronaphthalene‐1,4‐dione. This regioisomeric mixture was separated via column chromatography.

Intermediate **2** then underwent nucleophilic substitution with *N*‐BOC‐1,6‐diaminohexane in EtOH, producing regioisomers **3a** and **3b**. These isomers were formed in approximately equal amounts and were separated by chromatography on neutral alumina using ethyl acetate:hexane (3:7). The final HCl‐salt forms, **LHN‐1034** and **LHN‐1035**, were obtained through acid‐mediated BOC deprotection followed by salt formation.

### 3.2 Antibacterial Activity in Comparison With Conventional Antibiotics

The antimicrobial activity of **LHN‐1034** and **LHN‐1035** was compared with ciprofloxacin and tetracycline‐class antibiotics (Table 2). Minimum Inhibitory Concentrations (MICs) were determined after 48 h of incubation.

**TABLE 2:** Minimum Inhibitory Concentrations (MICs) of ciprofloxacin, tetracycline, oxytetracycline, LHN‐1034 and LHN‐1035 against tested microorganisms.

	Organism	Ciprofloxacin	Tetracycline	Oxytetracycline	LHN‐1034^a^	LHN‐1035^a^
Gram‐negative	*E. coli*	50 µM	50 µM	50 µM	> 200 µM	> 200 µM
	*S. typhimurium*	100 µM	100 µM	50 µM	> 200 µM	> 200 µM
	*A. baumannii*	> 200 µM	> 200 µM	100 µM	> 200 µM	> 200 µM
	*G. vaginalis*	100 µM	100 µM	> 200 µM	100 µM	50 µM
Gram‐positive	*C. acnes*	50 µM	50 µM	> 200 µM	100 µM	100 µM
	*E. faecalis*	25 µM	25 µM	50 µM	100 µM	> 200 µM
	*CCARM 3505 (QRSA)*	100 µM	100 µM	100 µM	100 µM	100 µM
	*CCARM 3506 (MRSA)*	50 µM	50 µM	> 200 µM	100 µM	50 µM
	*S. aureus (KCTC 3881)*	50 µM	50 µM	> 200 µM	50 µM	100 µM
	*S. aureus (CCARM 3155)*	> 200 µM	> 200 µM	100 µM	100 µM	100 µM

^a^ MIC values are expressed in μM. For LHN‐1034 and LHN‐1035, MIC values of 50 μM and 100 μM correspond to approximately 21.6 μg/mL and 43.3 μg/mL, respectively.

In Gram‐negative bacteria, ciprofloxacin exhibited MIC values of 50 µM for *E. coli* and 100 µM for *S. typhimurium*. Tetracycline and oxytetracycline also inhibited these strains. In contrast, **LHN‐1034** and **LHN‐1035** did not show MICs for *E. coli* or *S. typhimurium*. For *A. baumannii*, all tested compounds, including ciprofloxacin, showed MIC values of > 200 µM. Against *G. vaginalis*, both ciprofloxacin and **LHN‐1034** exhibited MIC values of 100 μM, whereas LHN‐1035 showed an MIC of 50 μM. **LHN‐1034** and **LHN‐1035** also inhibited drug‐resistant *S. aureus* strains. Against MRSA (CCARM 3506), **LHN‐1034** and **LHN‐1035** exhibited MIC values of 100 μM and 50 μM, respectively, whereas both compounds showed MIC values of 100 μM against QRSA (CCARM 3505).

Among Gram‐positive bacteria, *C. acnes* was susceptible to ciprofloxacin (MIC 50 µM) and both **LHN‐1034** and **LHN‐1035** (MIC 100 µM). *E. faecalis* was susceptible to ciprofloxacin (MIC 25 µM), but **LHN‐1035** showed no activity against this strain (> 200 µM). Notably, *S. aureus* (CCARM 3155) was resistant to ciprofloxacin, whereas **LHN‐1034** and **LHN‐1035** inhibited growth at 100 µM. Cell viability assays showed a concentration‐dependent effect on most bacterial strains (Figure 1). **LHN‐1034** reduced *C. acnes* viability from 96.9% at 6.25 µM to 14.2% at 50 µM. *S. aureus* strains also exhibited a decrease in viability with increasing concentrations. At 50 µM of **LHN‐1034**, viability was 41.8% for *S. aureus* (KCTC 3881), 40.5% for MRSA (CCARM 3506), 48.4% for QRSA (CCARM 3505), and 41.8% for *S. aureus* (CCARM 3155). *E. faecalis* viability was 42.5% at 50 µM. In contrast, *E. coli* viability was 76.8% at 50 µM.

**LHN‐1035** showed similar results. At 50 µM, the survival rates of *C. acnes* and MRSA (CCARM 3506) were 21.5% and 29.8%, respectively. *E. coli* showed resistance to **LHN‐1035**, consistent with the MIC data, with limited reduction in survival at 50 µM.

### 3.3 **LHN‐1034** and **LHN‐1035** Induce ROS Generation in Bacterial Cell

**FIGURE 2:**
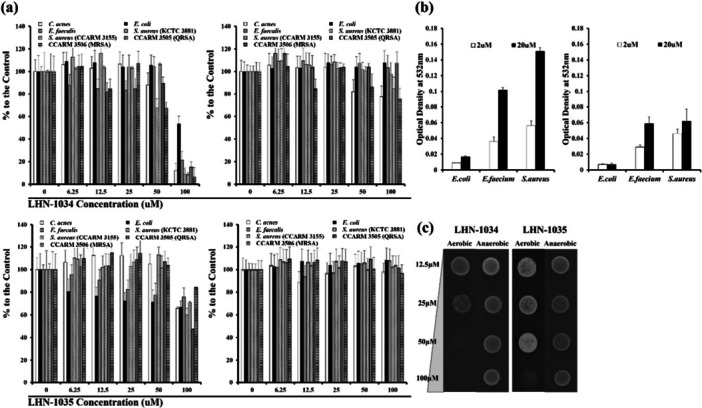
The ROS inducing activity of **LHN‐1034** and **LHN‐1035** on Gram‐positive cells. (a) Comparison of bacterial cell growth under aerobic (left panels) and anaerobic (right panels) conditions after treatment with **LHN‐1034** (upper panels) or **LHN‐1035** (lower panels). (b) Intracellular malondialdehyde (MDA) levels measured after treatment with **LHN‐1034** (left panel) or **LHN‐1035** (right panel) (c) Spotting analysis of *S. aureus* after treatment with each compound.

Bacterial growth was evaluated under aerobic and anaerobic conditions using OD_600_ measurements (Figure 2a). **LHN‐1034** and **LHN‐1035** exhibited concentration‐dependent antibacterial activity under aerobic conditions. **LHN‐1034** inhibited growth at tested concentrations, while **LHN‐1035** showed efficacy at 100 µM. Both compounds reduced the growth of *C. acnes* and *S. aureus* strains to less than 20% compared to the control. In contrast, under anaerobic conditions, inhibitory activity was reduced, and concentration‐dependent effects were not observed. **LHN‐1035** showed minimal effects on all tested bacteria under anaerobic conditions. *E. coli* was resistant to both compounds regardless of oxygen presence.

To assess ROS induction in Gram‐positive bacteria, malondialdehyde (MDA) levels were measured (Figure 2b). Treatments were performed at 2 µM or 20 µM. In *E. faecalis* and *S. aureus*, MDA levels increased in a concentration‐dependent manner. For *E. faecalis* and *S. aureus*, OD_532_ values were 0.036 and 0.056 at 2 µM, and 0.102 and 0.151 at 20 µM, respectively. MDA levels in *E. coli* did not increase significantly. Spotting analysis confirmed that antibacterial activity against *S. aureus* occurred only under aerobic conditions (Figure 2c). No growth differences were observed under anaerobic conditions in the presence of the compounds.

### 3.4 Cell Lysis Activity

**FIGURE 3:**
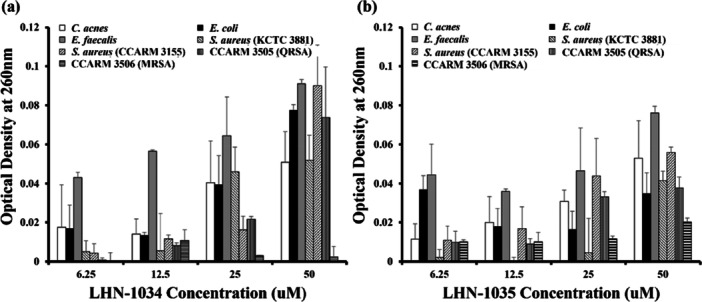
Amount of 260‐nm‐absorbing materials released from each bacterial strain after treatment with **LHN‐1034** or **LHN‐1035**. (a) Released intracellular components after treating **LHN‐1034**. (b) Released intracellular components after treating **LHN‐1035**.

To determine whether the compounds induce cell lysis, the release of 260 nm‐absorbing materials was measured (Figure 3). **LHN‐1034** induced a concentration‐dependent increase in the release of cellular contents from most bacterial strains. In *C. acnes*, absorbance at 260 nm reached 0.051 at 50 µM. Similar trends were observed for *E. coli* and *E. faecalis*, with values of 0.078 and 0.091 at 50 µM, respectively. Among *S. aureus* strains, leakage in *S. aureus* (KCTC 3881) increased from 0.006 at 6.25 µM to 0.052 at 50 µM. *S. aureus* (CCARM 3155) showed an increase from 0.004 to 0.090. *S. aureus* (CCARM 3505; QRSA) exhibited leakage of 0.074 at 50 µM, while *S. aureus* (CCARM 3506; MRSA) did not show leakage at the tested concentrations.

**LHN‐1035** also induced concentration‐dependent cell lysis. In *C. acnes*, absorbance ranged from 0.018 at 6.25 µM to 0.051 at 50 µM. *E. coli* exhibited minimal changes across all tested concentrations. *E. faecalis* showed an increase from 0.043 at 0 µM to 0.091 at 50 µM. In *S. aureus* (KCTC 3881) and *S. aureus* (CCARM 3155), values increased to 0.052 and 0.090 at 50 µM, respectively. *S. aureus* (CCARM 3505; QRSA) showed an increase from 0.001 at 6.25 µM to 0.074 at 50 µM. *S. aureus* (CCARM 3506; MRSA) exhibited leakage of 0.002 at 50 µM, representing a change compared to **LHN‐1034**.

### 3.5 Molecular Docking of **LHN‐1034** and **LHN‐1035** to the DNA Gyrase A‐Site

**FIGURE 4:**
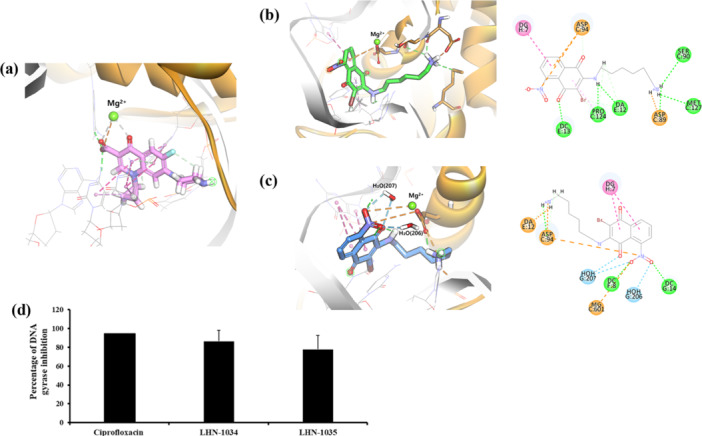
Molecular docking analysis of **LHN‐1034** and **LHN‐1035** at the DNA gyrase A‐site. (a) Reproduction of the co‐crystal binding pose of ciprofloxacin at the DNA gyrase A‐site (PDB ID: 5BTC), including the Mg^2+^‐mediated coordination network. (b) Predicted binding mode and key intermolecular interactions of **LHN‐1034** at the DNA gyrase A‐site. (c) Predicted binding mode and key intermolecular interactions of **LHN‐1035** at the DNA gyrase A‐site. (d) Inhibitory effects of **LHN‐1034**, **LHN‐1035**, and ciprofloxacin on *E. coli* DNA gyrase.

Molecular docking was performed to characterize the binding of **LHN‐1034** and **LHN‐1035** at the DNA gyrase A‐site using the *Mycobacterium tuberculosis* DNA gyrase A–DNA–ligand ternary complex (PDB ID: 5BTC) (Aldred et al. 2014). The binding pose of ciprofloxacin was reproduced to validate the protocol (Figure 4a). Ciprofloxacin exhibited π–π stacking interactions with DNA bases and an Mg^2+^‐mediated coordination network involving its carboxylate and keto groups (Blower et al. 2016).

**LHN‐1034** and **LHN‐1035** were predicted to bind within the DNA gyrase A‐site (Figure 4b, c). **LHN‐1034** formed π–π interactions with a DNA base (DG H:7) and engaged in hydrogen bonding and electrostatic interactions with Asp94, Asp89, Ser90, and Pro124. **LHN‐1035** exhibited a similar binding pose but showed involvement in the Mg^2+^‐centered metal–water interaction network. Its carbonyl oxygen atoms interacted with Mg^2+^ via bridging water molecules (HOH 206 and HOH 207).

Consensus docking indicated that **LHN‐1034** and **LHN‐1035** achieved LibDock scores of 113.96 and 112.41, respectively. These values were higher than the score for ciprofloxacin (96.84) under the same conditions (Diller and Merz 2001). **LHN‐1034** showed a slightly higher docking score compared to **LHN‐1035**.

### 3.6 DNA Gyrase Inhibition by **LHN‐1034** and **LHN‐1035**

To demonstrate the antibacterial mechanism of action, led by *in silico* predictions suggesting potential binding affinity to the enzyme, the inhibitory effects of **LHN‐1034** and **LHN‐1035** on *E. coli* DNA gyrase were evaluated (Figure 4d). The assay assessed the ability of the compounds to inhibit the ATP‐dependent supercoiling of relaxed plasmid DNA. Both derivatives exhibited great inhibitory activity against the enzyme at a concentration of 10 µM. **LHN‐1034** inhibited gyrase activity by 86.72 ± 11.14%, while **LHN‐1035** showed an inhibition rate of 77.96 ± 14.64%. Ciprofloxacin inhibited DNA gyrase by 94.29 ± 0.08% under the same conditions.

### 3.7 Computational Toxicity Assessment

Cytotoxicity was evaluated through in silico toxicity prediction (Table 3) (Banerjee et al. 2018; Pires et al. 2015). The predictions indicated that both compounds were strongly classified as skin irritants (*p* ≈ 0.98), whereas skin sensitization was predicted as non‐sensitizer (*p* ≈ 0.71), suggesting a low likelihood of allergic reactions. In addition, ocular irritation was predicted with very high probability (*p* ≈ 0.9998), highlighting the need to avoid ocular exposure in formulation development.

**TABLE 3:** In silico toxicity predictions relevant to topical antimicrobial use of LHN‐1034 and LHN‐1035.

Parameter	LHN‐1034	LHN‐1035
Skin Irritation	Irritant (*p* ≈ 0.98)	Irritant (*p* ≈ 0.98)
Skin Sensitization	Non‐sensitizer (*p* ≈ 0.71)	Non‐sensitizer (*p* ≈ 0.71)
Ocular Irritation	Irritant (*p* ≈ 0.9998)	Irritant (*p* ≈ 0.9998)
Human Intestinal Absorption	Very low (score ≈ 0.03, unitless)	Very low (score ≈ 0.03, unitless)
Skin Permeability–related Properties (logP, PSA)	logP ≈ 1.1, PSA ≈ 122 Å^2^	logP ≈ 1.1, PSA ≈ 122 Å^2^

Predicted pharmacokinetic parameters showed that human intestinal absorption (HIA) was low (≈ 0.03), indicating that these compounds are more suitable for topical formulations rather than systemic administration (Bos and Meinardi 2000). Moreover, logP (≈ 1.1) and PSA (≈ 122 Å^2^) values suggest limited skin permeability, supporting the possibility that the compounds may remain localized in the epidermis with minimal systemic exposure.

## 4 Discussion

The structural assignments of **LHN‐1034** (8‐nitro regioisomer) and **LHN‐1035** (5‐nitro regioisomer) were confirmed through ^1^H–^13^C (HMBC) analysis and comparison with related naphthoquinone regioisomers (Blackburn 2005). The formation of regioisomers in approximately equal proportions indicates that the nitro group at the C‐5 position does not exert a directing effect on the incoming amine at the C‐2 or C‐3 positions under the utilized reaction conditions. The results show that **LHN‐1034** and **LHN‐1035** possess selective activity against Gram‐positive bacteria. This selectivity results from structural differences between bacterial types, as the outer membrane of Gram‐negative bacteria functions as a permeability barrier that prevents many compounds from reaching intracellular targets (Nikaido 2003).

The efficacy of both compounds against multidrug‐resistant strains, including *S. aureus* (CCARM 3155), was observed. The finding that MIC values were lower than those of ciprofloxacin against the resistant strain indicates potential for antimicrobial therapy (Zang et al. 2022). This suggests that the mechanism of action for **LHN‐1034** and **LHN‐1035** is distinct from fluoroquinolones and tetracyclines, allowing them to bypass specific resistance mechanisms.

While determining the minimum bactericidal concentration (MBC) provides valuable information regarding the absolute bactericidal endpoint, the WST‐8 based cell viability assay was employed in this study to quantitatively evaluate the dose‐dependent metabolic inhibition of the bacterial cells. This colorimetric method enables a sensitive, high‐throughput assessment of cellular dehydrogenase activity, providing a profile of early metabolic impairment prior to complete cell death (Ishiyama et al. 1993). The reduction of antibacterial activity under anaerobic conditions indicates an oxygen‐dependent mechanism (Ahmad et al. 2015). This efficacy is characteristic of redox‐cycling agents. The naphthoquinone scaffold accepts electrons from cellular reductases to form radical intermediates, which transfer electrons to O_2_, generating superoxide anions and regenerating the parent quinone (Barrett et al. 1993). This cycle leads to the accumulation of ROS, which targets cellular components including DNA, proteins, and lipids (Spencer and Panda 2023). The TBARS assay supports this mechanism, as the concentration‐dependent increase in MDA indicates that the compounds induce oxidative stress. The low ROS induction in *E. coli* is consistent with its resistance, suggesting that these derivatives do not interact with the electron transport chain in Gram‐negative bacteria.

The release of 260 nm‐absorbing materials into the supernatant serves as an indicator of membrane damage and subsequent cell lysis. The concentration‐dependent increase in absorbance indicates that **LHN‐1034** and **LHN‐1035** induce a loss of membrane integrity. This lytic activity results from oxidative stress induced by the compounds (Dwyer et al. 2014). ROS induce lipid peroxidation, which destabilizes the phospholipid bilayer and causes membrane rupture (Repetto et al. 2012). This process leads to the release of cytoplasmic contents, such as nucleic acids, which absorb at 260 nm. These results suggest a dual mechanism of action involving intracellular killing by ROS and physical disruption of the cell membrane (Vatansever et al. 2013). Such mechanisms are useful for antimicrobial development as they increase the barrier to the development of bacterial resistance (Oldfield and Feng 2014).

The structural conservation of the DNA gyrase A‐site across bacterial species makes 5BTC a suitable model for the analysis of new antibacterial compounds (Aldred et al. 2014). The reproduction of the ciprofloxacin binding mode confirms the reliability of the docking procedure, as the interactions are consistent with previously reported complex structures (Blower et al. 2016). Docking results provide structural support for the antibacterial activity, showing that both compounds occupy the fluoroquinolone‐binding A‐site through aromatic stacking with DNA bases and interactions with conserved residues. The higher docking score and enzymatic inhibition observed for **LHN‐1034** suggest that the positional variation of the nitro group may optimize binding within the A‐site pocket. However, docking scores are interpreted as relative indicators of binding stability rather than direct measures of biological potency (Kitchen et al. 2004).

The inhibition of DNA gyrase activity contributes to the antibacterial efficacy of **LHN‐1034** and **LHN‐1035** (Zang et al. 2022). While fluoroquinolones primarily inhibit DNA gyrase, these derivatives exhibit a multimodal mechanism. The combination of gyrase inhibition, ROS induction, and membrane disruption overcomes resistance mechanisms associated with traditional antibiotics (Miethke et al. 2021; Suller and Lloyd 2002). This action impairs bacterial survival by simultaneously targeting the cell membrane, inducing oxidative stress, and blocking DNA replication (Belorgey et al. 2013; Silver 2007). This multi‐targeting approach reduces the probability of bacteria adapting to multiple targets (Juan et al. 2021).

The MTT assay is often unreliable for quinone derivatives because these compounds can interfere with tetrazolium reduction, leading to artifacts (Zhang et al. 2021). Therefore, in silico models were used to assess the safety profile. The low HIA values indicate that **LHN‐1034** and **LHN‐1035** are more suited for topical formulations than systemic administration (Bos and Meinardi 2000). Furthermore, the logP and PSA values suggest limited skin permeability, indicating that the compounds may remain localized in the epidermis and minimize systemic exposure. Although the in silico models predicted that **LHN‐1034** and **LHN‐1035** might possess strong skin‐irritating properties, their very low HIA and limited skin permeability make systemic administration unfeasible, positioning them exclusively as candidates for topical development. Furthermore, potential local irritation can often be effectively mitigated in clinical settings by employing advanced formulation strategies, such as encapsulation in liposomes, hydrogels, or nanoparticle‐based delivery systems, which control drug release and minimize contact toxicity (Gao et al. 2014).

In conclusion, this study demonstrates that **LHN‐1034** and **LHN‐1035** possess antibacterial activity against Gram‐positive bacteria, including multidrug‐resistant strains such as MRSA and QRSA. The mechanism of action is determined to be multimodal, involving ROS‐induced oxidative stress, disruption of membrane integrity, and inhibition of DNA gyrase activity. This multi‐targeting mechanism may lower the likelihood of resistance development. While activity against Gram‐negative bacteria was limited, the performance against Gram‐positive pathogens indicates that these 1,4‐naphthoquinone derivatives are lead compounds for topical application. Future research should focus on structural optimization and the evaluation of safety and efficacy *in vivo*.

## Author Contributions

**HyunChan Song:** conceptualization, methodology, writing – original draft, formal analysis. **Haena Lee:** conceptualization, methodology, writing – original draft, formal analysis. **Ji‐Hyun Yoon:** investigation, formal analysis. **Hyunkyung Choi:** conceptualization, writing – review and editing, supervision. **Ki‐Young Kim:** conceptualization, writing – review and editing, supervision. **Hakwon Kim:** writing – review and editing, supervision, resources.

## Conflicts of Interest

The authors declare no conflicts of interest.

## Supporting information


**Figure S1:** Chemical structure of YM155 and C5.

## Data Availability

The data that support the findings of this study are available from the corresponding author upon reasonable request.
