# Supplementary material for: Two Novel 1,4‐Naphthoquinone Derivatives as Potent Agents Against Multidrug‐Resistant Staphylococcus aureus and Pathogenic Gram‐Positive Bacteria
Source: Drug Dev Res. 2026 Jul 20;87(5):e70353. doi: 10.1002/ddr.70353 (PMC13382487; doi:10.1002/ddr.70353)

**Supplementary figure legends**

**Figure S1.** Chemical structure of YM155 and C5. (a) Chemical structure of YM155. (b) Chemical structure of C5.

**Figure S1.** Chemical structure of YM155 and C5

1. Chemical structure of YM155


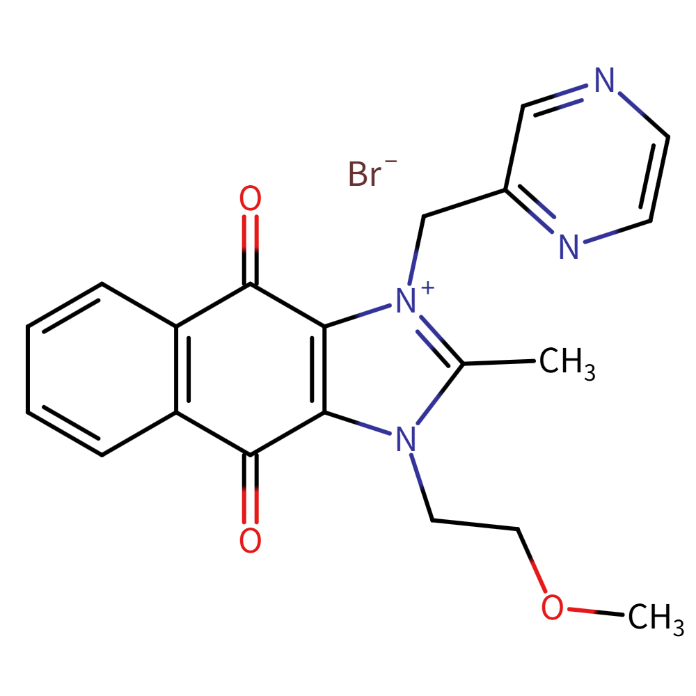


1. Chemical structure of C5


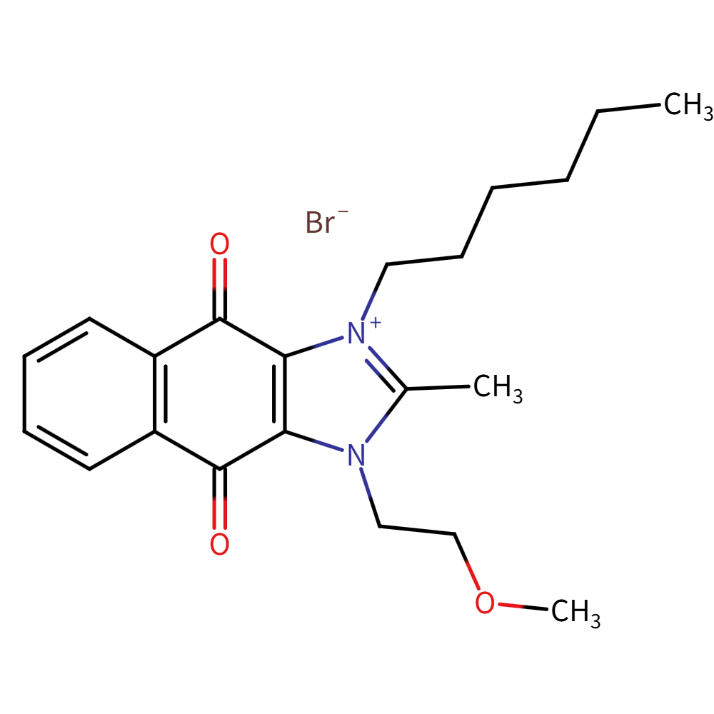

Supplement: Supplementary file 1 — Figure S1: Chemical structure of YM155 and C5. [file DDR-87-e70353-s001.docx]
